# Female gender, dissatisfaction with weight, and number of IBD related surgeries as independent risk factors for eating disorders among patients with inflammatory bowel diseases

**DOI:** 10.1186/s12876-022-02526-0

**Published:** 2022-10-17

**Authors:** Gianna Stoleru, Andrew Leopold, Amanda Auerbach, Shelley Nehman, Uni Wong

**Affiliations:** 1 University of Maryland Medical Center, 22 S Greene Street, Suite N3W42, Baltimore, MD 21201 USA; 2 University of Maryland School of Medicine, 22 S Greene Street, Suite N8E28, Baltimore, MD 21201 USA; 3 VA Maryland Healthcare System, 22 S Greene Street, Suite N8E28, Baltimore, MD 21201 USA

**Keywords:** Inflammatory bowel diseases, Eating disorders, EAT-26, Crohn’s disease, Ulcerative colitis

## Abstract

**Background:**

The prevalence and risk factors of eating disorders among patients with IBD are poorly described in existing literature. Early recognition and intervention may influence clinical outcomes in both physical and mental health. The primary aims of this study were to describe the prevalence and identify risk factors for eating disorders among patients with IBD using a validated questionnaire, the EAT-26.

**Methods:**

The EAT-26 was administered via email as an anonymous, unpaid, online survey to 1589 patients with an electronic medical record coded diagnosis of IBD (ulcerative colitis or Crohn’s disease) who had visited our Digestive Health Center in the last 3 years. Demographics and IBD characteristics were also included in our survey. A score of 20 or higher on the EAT-26 portion of the survey was considered a positive screen for eating disorder risk.

**Results:**

Fifteen (4.8%) survey participants screened positively for ED risk. These 15 participants who screened positively had statistically significant differences in self-identified gender (93% female, p = 0.031), happiness with current weight (80% dissatisfied with their current weight and trying to lose weight, p < 0.01), prior eating disorder diagnosis (20%, p < 0.01), and number of IBD related surgeries (27% having 3 or more, p = 0.013).

**Conclusions:**

This study identifies independent risk factors for eating disorder risk in patients with IBD including female gender, dissatisfaction with current weight, number of IBD related surgeries, and history of prior eating disorder diagnosis.

**Supplementary Information:**

The online version contains supplementary material available at 10.1186/s12876-022-02526-0.

## Background

Patients with inflammatory bowel diseases (IBD), namely Crohn’s disease (CD) and ulcerative colitis (UC), are at risk for behavioral health disorder such as eating disorders (ED) that include anorexia nervosa (AN), bulimia nervosa (BN) and binge eating. The prevalence and risk factors of ED among patients with IBD are poorly described in the existing literature. Early recognition and intervention may influence clinical outcomes in both physical and mental health. The primary aims of this study were to describe the prevalence and identify risk factors for ED among patients with IBD seen at a single tertiary referral center using a validated questionnaire, the Eating Attitudes Test-26 (EAT-26).

ED can reliably be diagnosed utilizing the *Diagnostic and Statistical Manual of Mental Disorders –* Fifth Edition (DSM V). While the list of diagnosable ED is constantly evolving, commonly recognized and distinct ED described in the DSM V include AN, BN, and avoidant/restrictive food intake disorder (ARFID) [1]. The majority of existing literature on ED comes from adolescent cohorts. In general, ED are described at a rate of 3.8% in female and 1.5% in male adolescents. These rates are even higher in adolescent populations with chronic illnesses such as type 1 diabetes mellitus, occurring in up to 37.9% in females and 15.9% in males [2]. In adults, up to 4% of women and 0.7% of men self-reported diagnosis of AN and/or BN [3].

Patients with ED have an increased risk of suicide and increased overall mortality when compared to the general population. One longitudinal study showed that even with access to treatment, the hazard ratios for all-cause mortality of AN was 6.51 (95% CI 3.46–12.26) and of BN was 2.97 (CI 1.90–4.65) [4]. While the most frequent causes of death from medical conditions in people with AN are circulatory collapse, cachexia and organ failure [5], suicide makes up a significant proportion of death in this population; as many as one in five people with AN who have died committed suicide [6]. It is crucial for medical providers to identify patients at risk for ED early and provide mental health resources and inter-professional referral in order to mitigate these risks.

ED behaviors have been studied in patients with other chronic conditions with primarily gastrointestinal symptomology, such as celiac disease or type 1 diabetes mellitus [7, 8] though literature on this topic in patients with IBD is scarce. While ED in patients with IBD has mostly been described in case reports [9], prevalence is poorly described and likely underdiagnosed. This may be, in part, due to a previously described phenomenon wherein the presenting symptoms of IBD were met with food avoidance, diet alteration and weight loss of the patient, leading to diagnostic confusion and delay of appropriate treatment [10]. A 2017 review of existing case reports found that comorbid ED and IBD are most commonly reported in young women, with CD and AN being the most commonly reported comorbid conditions [9]. Patients with IBD and ED have been reported to perpetuate weight loss by declining IBD therapies due to fear of weight gain [9]. Additionally, the weight gain from treatments like corticosteroids has been linked to the development or exacerbation of ED in patients with previously diagnosed IBD [11]. Yet another confounding factor in making the diagnosis of ED in patients with IBD is the increased cytokine release in active disease states affecting hunger and satiety signaling, leading to weight loss and anorexia outside of a diagnosable ED [12].

## Methods

We created an anonymous online survey, accessible by URL link with single use capability. The survey took an average of 5 min to complete. Participants were not required to answer every question. The final portion of the survey included the EAT-26 questionnaire. Screening for patients at risk for ED has been validated using the EAT-26 questionnaire [13]. The original questionnaire was proposed as a 40 item questionnaire in 1979, and was then simplified to the 26 item version in 1982. Demographics and IBD characteristics were also included in our survey. A score of 20 or higher on the EAT-26 portion of the survey was considered a positive screen for patient risk of ED. A copy of the survey in its entirety can found in the Additional file 1.

Our health system’s information technology team curated a list of patients with electronic medical record (EMR) coded diagnoses of IBD including CD and UC, who had visited our Digestive Health Center in the last 3 years. Only patients with a current email address in the EMR could be included in the study. The survey link was emailed to 1589 patients. All patients were emailed the survey link on two occasions, at an approximate 1 month interval.

IBM SPSS Statistics for Windows, Version 21.0 (IBM Corp., Armonk, N.Y., USA) was used for data analysis. Univariate logistic regression analysis was performed to identify statistically significant independent variables. Further multivariate binary logistic regression analysis confirmed statistical significance.

This study was reviewed by an IRB analyst at the University of Maryland and was determined to be exempt under 45 CFR 46.101(b) from IRB review.

## Results

Three hundred and eight (308, 19%) participants completed the entire survey. Participants were mostly age 25–34 years (29%), female (64%), Caucasian (82%), had CD (57%), had no previously diagnosed ED (97%), were unhappy with their current weight and trying to lose weight (43%), were diagnosed with IBD > 10 years ago (52%), had never been hospitalized for IBD (34%), had never undergone surgery for IBD (61%), were in remission by Manitoba Index (25%), took 1–3 medications (51%), and had an average BMI of 27 ± 7.3 kg/m^2^ (Table 1 and Additional file 2: Table S1).

**Table 1:** Demographic data

		EAT 26 screen negative (n = 293)	EAT 26 screen positive (n = 15)	Statistical test; p-value
Age	18–24	26	1	Fisher’s exact; p = 0.83
	25–34	83	6	
	35–44	75	4	
	45–54	41	3	
	55–64	43	1	
	65 +	25	0	
Self-identified gender	Female	182	14	Fisher’s exact; p = 0.031
	Male	110	1	
	Non-binary	1	0	
Race	White	239	13	Fisher’s exact; p = 0.144
	Hispanic	7	0	
	Black	24	1	
	Asian	21	0	
	Pacific islander	0	1	
	Other	2	0	
Type of IBD	CD	164	10	Fisher’s exact; p = 0.272
	UC	91	2	
	Indeterminate	38	3	
BMI kg/m^2^ (mean ± standard deviation)		30.9 ± 11.6	26.9 ± 6.91	Simple logistic regression; p = 0.045, R^2^ = 0.034

Comparison of demographic data for patients with IBD that screened positively for ED risk compared to those that screened negatively for ED risk by EAT-26

Fifteen (4.8%) survey participants screened positively for ED risk. Fourteen (93%) were female (p = 0.031), 6 (40%) were age 25–34 years (p = 0.83), and 10 (66.67%) had CD rather than UC (p = 0.272) using Fisher’s exact test. The 15 participants who screened positively had statistically significant differences in satisfaction with current weight (80% dissatisfied with their current weight and trying to lose weight, p < 0.01) (Fisher’s exact test), prior ED diagnosis (20%, p < 0.01) (Fisher’s exact test), and number of IBD related surgeries (27% having 3 or more, p = 0.013) using a one-way ANOVA (Table 2).

**Table 2:** Statistically significant survey results

Survey question	Survey answer	EAT 26 screen negative (n = 293)	EAT 26 screen positive (n = 15)	Univariate Statistical Test	Multivariate binary logistic analysis
Are you happy with your current weight?	Yes	109	1	Fisher’s Exact Test p < 0.01	p = 0.014
	No; not trying to gain or lose weight	36	0		
	No; trying to lose weight	123	12		
	No; trying to gain weight	25	2		
Number of IBD-related surgeries	0	184	4	Analysis of variance (ANOVA) p = 0.013	p = 0.023
	1	44	5		
	2	30	2		
	3 or more	34	4		
Prior ED diagnosis	No	288	12	Fisher’s Exact test p < 0.01	p = 0.01
	Yes	5	3		

Comparison of survey answers between those that screened positively for ED risk compared to those that screened negatively for ED risk by EAT-26 that are statistically significant

Multivariate binary logistic regression analysis confirmed statistical significance for IBD related surgeries (odds ratio (OR) = 1.78; confidence interval (CI) = 1.08–2.9; p = 0.023), dissatisfaction with current weight (OR = 2.9; CI = 1.2–6.3; p = 0.014), and prior ED diagnosis (OR = 13.2; 1.8–94.6; p = 0.01) (Table 2). Also incorporated into the multivariate logistic regression were age (p = 0.092), race (0.268), BMI (p = 0.341), and gender (p = 0.073). The sensitivity of EAT-26 screening was 99.7%, with a specificity of 20%, yielding an overall classification accuracy rate of 95.8%.

## Discussion

Our findings are underscored by existing literature. A smaller study of 83 patients found 16% of survey participants screened positively on the EAT-26 questionnaire [14]. Another study identified female gender and body dissatisfaction as independent risk factors for ED in patients with type 1 diabetes mellitus [2]. Additionally, it is known that patients with a history of IBD-related surgery have poorer body image and cosmesis [15].

This study identifies potential risk factors for ED in patients with IBD seen at a tertiary referral center. Independent risk factors for ED in IBD patients include female gender, dissatisfaction with current weight, number of IBD related surgeries, and history of prior ED diagnosis. Observational, but not statistically significant, associations were found between younger age and higher disease burden or activity by Manitoba Index.

This study provides important data on ED in patients with IBD using a well-known, previously validated tool. A discrete and anonymous online survey was used to increase patient comfort and participation. The number of participants in this survey study is much larger compared to existing literature [14].

There are several limitations to this study. This was a one center study with a 19% completion rate. As such, the survey is susceptible to nonresponse bias in the subset of participants who completed the survey may differ substantially from the participants who did not complete the survey. Additionally, patients with IBD managed by providers in an academic institution may infer worse or more resistant disease compared to patients with IBD managed in the community practice setting, who were not included in this study. The survey respondents were mostly age 25–34 years (29%), female (64%) and Caucasian (82%), which may not be generalizable. Due to limited response and self-selection bias in this voluntary survey study, true prevalence cannot be established. The multivariate regression model provided for a high sensitivity, but low specificity. Incorporating the EAT-26 survey provides for a low number of false negatives, only 1 patient in this study. The high sensitivity of employment of the EAT-26 makes it a useful screening tool to determine which patients should be referred for expert evaluation. The low specificity of this model does however lead to some false positives (12). Therefore, results of the EAT-26 survey should be interpreted with caution and with the assistance of a trained specialist in eating disorders to determine the clinical relevance and implications of their screen.

## Conclusions

In conclusion, our study identifies independent risk factors for ED in patients with IBD including female gender, dissatisfaction with current weight, number of IBD related surgeries, and history of prior ED diagnosis. To our knowledge, this is the first paper describing independent risk factors for ED in patients with IBD. Future investigation to examine the true prevalence may include a standardized protocol to screen all IBD patients for ED using the EAT-26 questionnaire during clinical visits. Knowing the true prevalence and risk factors for ED in patients with IBD will guide screening practices in the outpatient setting and promote early inter-professional referral for best outcomes.

## Supplementary Information

**Additional file 1.** Full study survey.

**Additional file 2.** Full survey results.

## Data Availability

The datasets used and/or analyzed during the current study are available from the corresponding author on reasonable request.

## Abbreviations

IBD: Inflammatory bowel diseases
CD: Crohn’s disease
UC: Ulcerative colitis
ED: Eating disorders
AN: Anorexia nervosa
BN: Bulimia nervosa
EAT-26: Eating-attitudes test-26
EMR: Electronic medical record
