# Supplementary material for: Female gender, dissatisfaction with weight, and number of IBD related surgeries as independent risk factors for eating disorders among patients with inflammatory bowel diseases
Source: BMC Gastroenterol. 2022 Oct 17;22:438. doi: 10.1186/s12876-022-02526-0 (PMC9578268; doi:10.1186/s12876-022-02526-0)
Supplement: Supplementary file 1 — Additional file 1. Full study survey. [file 12876_2022_2526_MOESM1_ESM.pdf]

Eating Attitudes in Patients with IBD

1. Age

2. What was your gender at birth?

- Female
- Male
- Prefer not to say
- Other (please specify)

3. What is your current weight (in pounds)?

4. What is your lowest weight (in pounds)?

5. What is your highest weight (excluding pregnancy, in pounds)?

6. What is your ideal weight (in pounds)?

7. What is your height (in feet ' and inches '')?

8. Are you happy with your current weight?

- ◯ Yes

- ◯ No, I am trying to gain weight.
- ◯ No, I am trying to lose weight.
- ◯ No, but I am not actively trying to gain or lose weight at this time.

9. Race

[∨]

10. Marital status

- ◯ Single
- ◯ Not married but in a relationship
- ◯ Married
- ◯ Divorced
- ◯ Widowed

11. Household income

[∨]

12. Highest level of education

- ◯ Did not complete high school

- Graduated high school
- Technical or occupational certificate
- Some college
- College degree
- Graduate degree
- Doctoral degree

13. Zip code

14. How many hours per week do you work, volunteer and/or attend classes?

- 40 hours or more
- 20-40 hours
- Less than 20 hours
- I am not in school, working, or volunteering at this time

15. Type of Inflammatory Bowel Disease

- Crohn's disease

- ◯ Ulcerative colitis
- ◯ IBD, indeterminate

16. When were you first diagnosed with IBD?

- ◯ Within last 5 years
- ◯ 5-10 years ago
- ◯ More than 10 years ago

17. How many surgeries have you had for your IBD?

- ◯ 0
- ◯ 1
- ◯ 2
- ◯ 3 or more

18. How many hospitalizations have you had for gastrointestinal (GI) symptoms?

- ◯ 0
- ◯ 1 - 2
- ◯ 3 - 5
- ◯ 5 - 10

◯ More than 10

19. Which medication(s) are you CURRENTLY taking for your IBD? (check all that apply)

☐ Remicade/Inflectra

☐ Humira

☐ Cimzia

☐ Simponi

☐ Entyvio

☐ Stelara

☐ Azathioprine/6MP

☐ Methotrexate

☐ Xeljanz

☐ Mesalamine

☐ Prednisone/Budesonide/Uceris

☐ None

Other (please specify)

20. Over the past 6 months, how often did you have symptoms related to your IBD?

- ◯ I was well in the past 6 months - what I consider remission
- ◯ Rarely active, giving me symptoms only a few days of the past 6 months
- ◯ Occasionally active, giving me symptoms 1-2 days per month
- ◯ Sometimes active, giving me symptoms on some days
- ◯ Often active, giving me symptoms on most days
- ◯ Constantly active, giving me symptoms every day

21. How many medications do you take for ALL of your medical condition(s)? (please include any non daily injections/infusions in your count)

- ◯ 0
- ◯ 1-3
- ◯ 4-6
- ◯ 7-10
- ◯ 11+

22. Do you currently smoke cigarettes or not?

- ◯ Yes, I do
- ◯ No, I do not

23. Have you ever been diagnosed with an eating disorder? (check all that apply)

- ☐ No
- ☐ Yes, binge eating disorder
- ☐ Yes, bulimia nervosa
- ☐ Yes, anorexia nervosa

EAT-26 Questionnaire

24. I am terrified about being overweight

- ◯ Always
- ◯ Usually
- ◯ Often
- ◯ Sometimes
- ◯ Rarely

- Never

25. I avoid eating when I am hungry

- Always
- Usually
- Often
- Sometimes
- Rarely
- Never

26. I find myself preoccupied with food

- Always
- Usually
- Often
- Sometimes
- Rarely
- Never

27. I have gone on eating binges where I feel that I may not be able to stop

- ◯ Always
- ◯ Usually
- ◯ Often
- ◯ Sometimes
- ◯ Rarely
- ◯ Never

28. I cut my food into small pieces

- ◯ Always
- ◯ Usually
- ◯ Often
- ◯ Sometimes
- ◯ Rarely
- ◯ Never

29. I am aware of the calorie content of foods that I eat

- ◯ Always
- ◯ Usually

- Often
- Sometimes
- Rarely
- Never

30. I particularly avoid foods with high carbohydrate content (i.e. bread, rice, potatoes, etc.)

- Always
- Usually
- Often
- Sometimes
- Rarely
- Never

31. I feel that others would prefer if I ate more

- Always
- Usually
- Often
- Sometimes
- Rarely

- Never

32. I vomit after I have eaten

- Always
- Usually
- Often
- Sometimes
- Rarely
- Never

33. I feel extremely gulity after eating

- Always
- Usually
- Often
- Sometimes
- Rarely
- Never

34. I am preoccupied with a desire to be thinner

- Always
- Usually
- Often
- Sometimes
- Rarely
- Never

35. I think about burning up calories when I exercise

- Always
- Usually
- Often
- Sometimes
- Rarely
- Never

36. Other people think that I am too thin

- Always
- Usually

- Often
- Sometimes
- Rarely
- Never

37. I am preoccupied with the idea of having fat on my body

- Always
- Usually
- Often
- Sometimes
- Rarely
- Never

38. I take longer than others to eat my meals

- Always
- Usually
- Often
- Sometimes
- Rarely

- Never

39. I avoid foods with sugar in them

- Always
- Usually
- Often
- Sometimes
- Rarely
- Never

40. I eat diet foods

- Always
- Usually
- Often
- Sometimes
- Rarely
- Never

41. I feel that food controls my life

- Always

- Usually
- Often
- Sometimes
- Rarely
- Never

42. I display self-control around food

- Always
- Usually
- Often
- Sometimes
- Rarely
- Never

43. I feel that others pressure me to eat

- Always
- Usually
- Often
- Sometimes
- Rarely

- Never

44. I give too much time and thought to food

- Always
- Usually
- Often
- Sometimes
- Rarely
- Never

45. I feel uncomfortable after eating sweets

- Always
- Usually
- Often
- Sometimes
- Rarely
- Never

46. I engage in dieting behavior

- Always

- Usually
- Often
- Sometimes
- Rarely
- Never

47. I like my stomach to be empty

- Always
- Usually
- Often
- Sometimes
- Rarely
- Never

48. I have the impulse to vomit after meals

- Always
- Usually
- Often
- Sometimes
- Rarely

◯ Never

49. I enjoy trying new rich foods

◯ Always

◯ Usually

◯ Often

◯ Sometimes

◯ Rarely

◯ Never

Done

Powered by
SurveyMonkey®
See how easy it is to create a survey.

Privacy & Cookie Notice
