# Supplementary material for: Female gender, dissatisfaction with weight, and number of IBD related surgeries as independent risk factors for eating disorders among patients with inflammatory bowel diseases
Source: BMC Gastroenterol. 2022 Oct 17;22:438. doi: 10.1186/s12876-022-02526-0 (PMC9578268; doi:10.1186/s12876-022-02526-0)
Supplement: Supplementary file 2 — Additional file 2. Full survey results. [file 12876_2022_2526_MOESM2_ESM.docx]

*Supplementary Table 1.* Further Survey Results

| Survey question | Survey answer | EAT 26 screen negative (n=293) | EAT 26 screen positive (n=15) | p-value by Fisher’s exact test |
| --- | --- | --- | --- | --- |
| Marital status | married | 166 | 7 | p=0.692 |
|  | single | 71 | 1 |  |
|  | relationship | 34 | 1 |  |
|  | widowed | 4 | 0 |  |
|  | divorced | 18 | 1 |  |
| Income | <$15,000 | 18 | 3 | p=0.111 |
|  | $15,000-$29,999 | 12 | 0 |  |
|  | $30,000-$49,999 | 26 | 4 |  |
|  | $50,000-$74,999 | 33 | 11 |  |
|  | $75,000-$99,999 | 42 | 2 |  |
|  | $100,000-$150,000 | 83 | 2 |  |
|  | >$150,000 | 79 | 3 |  |
| Education level | Did not finish high school | 3 | 0 | p=0.989 |
|  | Graduated high school | 13 | 0 |  |
|  | Technical certificate | 10 | 0 |  |
|  | Some college | 40 | 2 |  |
|  | College degree | 103 | 7 |  |
|  | Graduate degree | 92 | 5 |  |
|  | Doctoral degree | 32 | 1 |  |
| Hours working or volunteering per week | None | 57 | 3 | p=1 |
|  | <20 | 20 | 1 |  |
|  | 20-40 | 43 | 2 |  |
|  | >40 | 173 | 9 |  |
| Time since diagnosis of IBD | <5 years prior | 66 | 4 | p=0.542 |
|  | 5-10 years prior | 76 | 2 |  |
|  | >10 years | 151 | 10 |  |
| Manitoba index | I was well within the past 6 months – what I consider remission | 76 | 2 | p=0.386 |
|  | Rarely active, giving me symptoms only a few days of the past 6 months | 49 | 2 |  |
|  | Occasionally active, giving me symptoms 1-2 days per month | 40 | 3 |  |
|  | Sometimes active, giving me symptoms on some days | 59 | 2 |  |
|  | Often active, giving me symptoms on most days | 40 | 2 |  |
|  | Constantly active, giving me symptoms every day | 29 | 4 |  |
| Number of medications | 0 | 15 | 1 | p=0.197 |
|  | 1-3 | 150 | 6 |  |
|  | 4-6 | 75 | 6 |  |
|  | 7-10 | 37 | 2 |  |
|  | >11 | 16 | 0 |  |
| Number of hospitalizations in the last year | 0 | 104 | 2 | p=0.386  (ANOVA) |
|  | 1-2 | 85 | 5 |  |
|  | 3-5 | 48 | 3 |  |
|  | 5-10 | 26 | 3 |  |
|  | >10 | 30 | 2 |  |

Comparison of additional survey answers between those that screened positively for ED risk compared to those that screened negatively for ED risk by EAT-26.
